# Editorial: Molecular mechanisms and new therapeutic targets in epithelial to mesenchymal transition (EMT) and fibrosis, volume II

**DOI:** 10.3389/fphar.2022.1008955

**Published:** 2022-09-06

**Authors:** Cecilia Battistelli, Marc Diederich, Timothy Joseph Keane, Pilar Sandoval, Sergio Valente, Raffaele Strippoli

**Affiliations:** ^1^ Department of Molecular Medicine, Sapienza University of Rome, Rome, Italy; ^2^ Department of Pharmacy, College of Pharmacy, Seoul National University, Seoul, South Korea; ^3^ Department of Materials, Imperial College London, London, United Kingdom; ^4^ Severo Ochoa Molecular Biology Center (CSIC-UAM), Madrid, Spain; ^5^ Department of Chemistry and Technologies of Drugs, Sapienza University of Rome, Rome, Italy; ^6^ Istituto Nazionale per le Malattie Infettive Lazzaro Spallanzani (IRCCS), Rome, Italy

**Keywords:** EMT, epigenetics, fibrosis, natural compound, non-coding RNAs, therapeutic targets

The induction of inflammatory epithelial-mesenchymal transition (EMT)/fibrosis requires a complex cellular reprogramming process involving epithelial, stromal, and immune cells and has important implications on cell survival, plasticity, and migratory/invasive abilities.

A wide array of extracellular stimuli, including soluble mediators, cell-to-cell interactions, and binding to the extracellular matrix (ECM), drive changes in resident cells towards a mesenchymal-like/profibrotic phenotype.

The classical EMT/fibrosis pathways induced by transforming growth factor (TGF)-β, the central profibrotic mediator, as well as by tumor necrosis factor (TNF)α and epidermal growth factor (EGF) are well known. Here we focused on the roles of new intracellular mechanisms involved in the modulation of EMT/fibrosis. Moreover, a better understanding of the crosstalk between classical EMT/fibrosis pathways and stress signaling pathways, such as autophagy and unfolded protein response (UPR) become increasingly important in the field.

Epigenetic processes, including histone acetylation and DNA/histone methylation, were shown to be essential modulators of the persistence of a new mesenchymal-like state or the reversion to an epithelial-like phenotype. Recent single-cell RNA-sequencing (scRNA-seq) experiments revealed that EMT transition is a transcriptional continuum of numerous epithelial-mesenchymal states rather than a binary epithelial vs. mesenchymal model. These recent insights considerably enhanced the understanding of the complexity and cell-specificity of this essential physio-pathological process.

However, despite considerable efforts in the field, new mechanisms remain to be elucidated, and cell-type specificities have yet to be fully characterized.

In this Research Topic, Sara Lovisa provided an updated overview of the recent debate on the definition of EMT, analyzing the impact of new technologies such as single cell transcriptomics. Moreover, the author summarized the different strategies used to define EMT in fibrotic disorders.

Brasier et al. analyzed the mechanisms leading to changes in pulmonary cell plasticity induced by aeroallergens and respiratory viruses, leading to pathological airway remodeling. In particular, the authors elucidated the complex interplay between the hexosamine pathway, UPR, the inflammatory inhibitor of IκB kinase (IKK)-nuclear factor (NF)-κB pathway, and the regulation of epigenetic changes mediated by the recruitment of bromodomain-containing protein (BRD)4.

Li et al. used an experimental model of murine silicosis to study the capacity of metformin, a biguanide antidiabetic drug against type 2 diabetes, to attenuate lung fibrosis. The authors also co-cultured human macrophages and human bronchial epithelial cells treated with silica particles and metformin. Mechanistically, the manuscript elucidates the effect of metformin on autophagy regulated by the AMP-activated protein kinase (AMPK) - mammalian target of rapamycin (mTOR) pathway, leading to reduced silica particle-induced fibrosis.

Liu et al. investigated the importance of autophagy in murine peritoneal fibrosis (PF). Autophagy inhibitor 3-methyl adenine (3-MA) alleviated PF by inhibiting EMT. The authors observed the activation of multiple EMT-related pathways such as TGF-β/mothers against decapentaplegic homolog (SMAD)3, epidermal growth factor receptor (EGFR)/extracellular regulated kinase (ERK)1/2, and transcription factors signal transducer and activator of transcription (STAT)3 and NF-κB. Moreover, inhibition of autophagy attenuated peritoneal angiogenesis in the injured peritoneum.

This Research Topic describes different pharmacological approaches to preventing PF. Kopytina et al. focused on peritoneal dialysis (PD) fluid biocompatibility in the genesis of PF. The choice of PD fluid can lead to fibrosis, and PF represents the main cause of PD discontinuation in patients with end-stage kidney disease. Kopytina et al. analyzed steviol glycoside (SG)-based fluids compared to high and low glucose-based dialytic fluids. SG-based PD fluids increased biocompatibility and reduced induction of mesothelial to mesenchymal transition (MMT), a mesothelium-specific form of EMT.

Xie et al. analyzed the protective role of the hormonal peptides ELABELA (ELA) and aplin in MMT. ELA is a polypeptide hormone secreted by the vascular endothelium and the kidneys. Aplin is another hormone known to have a protective effect on organ fibrosis. Both hormones were modulated in patients exposed to PD fluids.

Treatment with the active proprotein of 32 amino acids, ELA-32, reversed the TGF-β1-induced reduction of the epithelial cell markers and suppressed the expression of mesenchymal cell markers by inhibiting the phosphorylation of SMAD2/3, ERK1/2, and protein kinase B (AKT).

Liu et al. analyzed the role of histone deacetylase (HDAC)-6 in MMT. The authors describe that the HDAC6 inhibitor tubastatin or genetic silencing of HDAC6 maintained the expression of E-cadherin but suppressed mesenchymal gene expression. Accordingly, HDAC6 inhibition maintained the epithelial/mesothelial phenotype in mesothelial cells treated with interleukin (IL)-6. Mechanistically, tubastatin suppressed the expression of TGF-β receptor I (TGFβRI), the phosphorylation of SMAD3, and the activation of Janus kinase (JAK)2 and STAT3.

Another non-tumor fibrosis model studied in this Research Topic is kidney fibrosis. Kidney fibrosis results from a wide array of inflammatory insults implicating both stromal and immune responses.

Zhang et al. analyzed the effect of a combination of mycophenolate and rapamycin (MR) on kidney fibrosis in a murine lupus nephritis experimental system. Mycophenolate is an immunosuppressant drug, whereas rapamycin is a known mTOR inhibitor. The authors found that the combination of these drugs at reduced doses resulted in reduced glomerular sclerosis and tubular atrophy, amelioration of kidney dysfunction, and improved survival. MR treatment reduced the expression of TGF-β1, IL-6, α-smooth muscle actin (α-SMA), fibronectin, and collagen I and III.

Martinez-Salgado et al. analyzed the role of activin receptor-like kinase 1 (ALK1) in tissue fibrosis and angiogenesis in a murine model of unilateral ureteral obstruction (UUO). Through a heterozygous mouse experimental system, ALK1 was demonstrated to promote vascular rarefaction and maturation and the emergence of myofibroblasts of vascular origin.

He et al. focused on the physiopathology of tubular epithelial cells (TECs). These cells play a role in kidney fibrosis undergoing partial EMT (pEMT), characterized by co-expression of both epithelial and mesenchymal markers and production of extracellular mediators favoring fibrogenesis in the interstitium. The authors discussed how wingless-related integration site (Wnt)/β-catenin inhibition by natural compounds, specific inhibitors, or genetic intervention might attenuate tubular EMT and fibrosis.

Systemic sclerosis (SSc) is a multi-system rheumatic disease characterized by vascular dysfunction, autoimmune abnormalities, and progressive organ fibrosis.

Dai et al. summarized the current knowledge regarding immune and stromal cells in SSc patients discussing their potential roles in SSc pathogenesis and focusing on recent advances in identifying new cellular subtypes by scRNA-seq.

Overall, more profound knowledge of physio-pathological mechanisms controlling EMT dynamics and the validation of new pharmacological approaches will help develop future regenerative medicine strategies to improve the personalized control of EMT and fibrotic responses in specific and localized manners.

